# The Effect of Font Size on Reading Comprehension on Second and Fifth Grade Children: Bigger Is Not Always Better

**DOI:** 10.1371/journal.pone.0074061

**Published:** 2013-09-19

**Authors:** Tami Katzir, Shirley Hershko, Vered Halamish

**Affiliations:** 1 Edmond J. Safra Brain Research Center for the Study of Learning Disabilities & Department of Learning Disabilities and Special Education, University of Haifa, Haifa, Israel; 2 Institute of Information Processing and Decision Making, University of Haifa, Haifa, Israel; CSIC-Univ Miguel Hernandez, Spain

## Abstract

Research on reading development has focused on the linguistic, cognitive, and recently, metacognitive skills children must master in order to learn to read. Less focus has been devoted to how the text itself, namely the *perceptual* features of the words, affects children’s learning and comprehension. In this study, we manipulated perceptual properties of text by presenting reading passages in different font sizes, line lengths, and line spacing to 100 children in the second and fifth grades. For second graders (Experiment 1), decreasing font size, as well as increasing line length, yielded significantly lower comprehension scores. Line spacing had no effect on performance. For fifth graders (Experiment 2), decreasing font size yielded higher comprehension scores, yet there were no effects for line length and line spacing. Results are discussed within a "desirable difficulty" approach to reading development.

## Introduction

Consider the subjective experience of a second grader reading a text, poorly photocopied and written in a small font. In contrast, imagine her reading large print, centered on the page, and subjectively easy to read. Intuitively, one may think that these perceptual and typographical factors are only related to the child’s motivation to read and will not affect her comprehension. Very little research has focused on potential developmental effects of manipulating physical properties of print (e.g., print size, font type, etc.) [1-3]. In adults, for example, it has been found that altering text presentation to a less familiar format, hence making it less perceptually fluent (words in italics) led to better memory of studied material in adults and high school students [1]. Using a different manipulation of text, another study found that extra large letter spacing enhanced the performance of word reading in children with dyslexia [3]. Currently, due to scarce research, all that can be concluded is that the effects of altering text presentation may differ by the specific manipulation and by population. It may also affect different aspects of reading: rate, accuracy and comprehension. In this study, we focus on an understudied question which is, can a mere manipulation of perceptual features of text enhance reading comprehension among second and fifth grade children.

### Reading Development and Reading Comprehension

Developmental models of reading assume that reading is made up of component skills [4-6]. These components begin with letter-sound recognition and then proceed to decoding skills. While each component is sufficient for a time, new skills must be achieved if reading proficiency is to increase. Later components include the development of efficiency, comprehension, and the ability to integrate and synthesize materials.

Children are assumed to progress from learning about print itself to learning about the alphabet, sounds of the letters, and letter groups [4]. During the initial period of learning to decode, in the first and second grades, the reader is "glued to the print." Reading is slow and laborious, as new readers still receive many cues about how to decode words from the letters themselves. By reading material that has familiar content and language style, children develop the ability to use context to decipher words, as well as fluent and effortless reading. By fourth grade, children are expected to be efficient readers, reading rapidly, comprehending complex materials, and making inferences about the text [4,7,8]. A greater reliance on meaning is also evident. Characteristics of children in fourth grade and above include the ability to concentrate less on the print and more on details and ideas. This developmental shift has been referred to as a shift from *learning to read* to *reading in order to learn* [4].

The ultimate goal of reading development is efficient reading comprehension, defined as a process of extracting and constructing meaning through interaction and involvement with written language [9]. What factors determine reading comprehension? Empirical evidence demonstrates that phonological processing, rapid automatized naming, orthographic processing, and word identification [7], as well as IQ [10], memory and attention [11], and higher order processes [12], all predict a significant portion of the variance in reading comprehension. However, even when these measures are entered into regression models, much of the variance in reading comprehension remains unexplained [13].

These findings have resulted in a shift towards a multi-dimensional view of reading comprehension that goes beyond cognitive and linguistic processes. The RAND model of reading comprehension [14] suggests that in order to understand the complex process of comprehension there is a need to concurrently examine a triangulation of the contribution of reader characteristics, text type, and environmental factors [13,15,8,9]. Interestingly, while much work has focused on the influence of the nature of the text type on reading (e.g., narrative vs. expository text) [14], very little work has focused on the typographical properties of text presentation. Could altering the perceptual features of the words, such as font size, line spacing, etc.’, actually lead to performance differences in reading comprehension? A central assumption in the reading comprehension literature is that in order to improve comprehension, the reader must improve his skills (e.g., phonological skill, vocabulary, decoding abilities, reading rate). However, what if comprehension can be improved by simply changing factors that are external to the reader, such as the typographical properties of the text, without changing its content? The current study was designed to address these questions.

### The Effects of Manipulating Perceptual Presentation on Cognitive Performance

Many education researchers believe that reducing extraneous cognitive load is always beneficial for the learning situation. If a student was able to learn new information easily, both the student and the teacher are likely to label the session as successful regardless of whether the student is able to retrieve the information later [16]. However, research in cognitive psychology suggests just the opposite. In many cases, the more challenging a learning session is, the better subsequent long term memory for the material studied in that session will be [17]. It may be that greater cognitive engagement leads to deeper processing, which then facilitates encoding and subsequent retrieval [18]. Thus, it has been found that the most effective learning strategies involve introducing difficulties for the learner. One clear example of a "desirable difficulty" [17,19] is the interleaving of to-be-learned materials, rather than blocking them, in a way that creates, at least temporarily, contextual interference for the learner [20]. Interleaving has been found to produce stronger learning than blocking, at least in the long run.

Yet another way to make learning more challenging is to manipulate disfluency, the subjective metacognitive experience of difficulty associated with cognitive tasks. Disfluency has been found to be strongly related to confidence in the ability to remember new information [21], with greater disfluency yielding lower confidence. In turn, when learners are less confident in how well they have learned the material, they are more likely to engage in more effortful and elaborative processing [22]. Indeed, disfluency has been shown to impact cognitive processing independently of actual cognitive difficulty (for example, the amount of material to be studied) [23]. For example, a recent study has demonstrated that creating disfluency by presenting words upside-down for study enhanced later recall for these words, compared to words that were presented right-side up [24]. It has also been shown that disfluency leads people to process information more carefully [25] and yields better oral comprehension [26]. Based on the above findings, we raise the following questions: Can manipulating perceptual features of text, which have been shown to create disfluency effects in adults, also lead to better reading comprehension in children? Will the effects of the manipulation depend on stages of reading development?

### The Developmental Effects of Manipulating Perceptual Presentation of Text

Manipulations of perceptual features of text build upon the assumption that the visual system makes use of relative size as a perceptual cue that conveys important information regarding the proximity of a stimulus [27]. Oppenhiemer and his colleagues manipulated perceptual presentation of text simply by adopting fonts that were more difficult to read [1,28] by choosing faded shades, small fonts, and unclear photocopying of text. A different way to manipulate text presentation and create text disfluency may be to manipulate the spacing between lines and line lengths, under the assumption that these changes pose greater challenge for readers [29].

Manipulating text presentation may affect reading rate and accuracy [29] as well as feeling of proficiency. Indeed, studies focusing on feelings of proficiency, mainly in adults, report that processing words presented in larger fonts was subjectively more fluent than processing words presented in smaller fonts [30,31]. Importantly, it has been found that simple interventions, such as presenting educational materials on PowerPoint slides and handouts in italics (which children are less accustomed to) as opposed to presenting the same text in a standard, non-italesized format, engaged both university and high school students in more elaborative processing and even subsequently resulted in improved educational outcomes, including higher grades [1]. In contrast, a study of word lists showed that font size did not affect recall [performance] among university students, though it did affect their judgment of learning, with larger fonts associated with greater estimations of remembrance [32]. In these studies the participants were all skilled readers that were passed the initial phases of reading development. However, the effect of such manipulations may be different for poor readers as well as for younger children.

Studies of adults that have taken into account variability in reading skills have found that manipulating text presentation has opposite effects on good and poor readers. Thus, increasing text difficulty by deleting letters led poor readers to show decreased recall, whereas good readers showed improved recall with letter deletion [33]. Based on these findings, we suggest that manipulating perceptual presentation of text might have differential effects on reading comprehension for skilled versus novice readers.

The majority of studies manipulating perceptual presentation of text in children have manipulated font size and line length and examined the effects on reading rate and accuracy and have not looked at its effect on reading comprehension. Interestingly, studies that examined font size found different effects for children at different stages of reading development: A relatively small font size was found to decrease the reading rates of five- to seven-year-olds, but had no effect on children in third to fifth grade [34]. Similarly, another study compared the reading rates of children with dyslexia in second through fourth grade and reading-level matched controls [35]. Dyslexic children benefited from larger fonts while their reading-level matched peers, similar to the results of college students previously described [32], showed no font size effects on reading rate and accuracy.

Regarding line length, in a study on six-year-olds, no differences were found in reading rate and accuracy between short and long lines, controlling for the number of words in a line [36]. However, another study found that large fonts were read as well as smaller fonts with large spacing between the words (which results in longer lines) [37]. Since there were no conclusive results across the two studies, and as line length and font size were concurrently manipulated in this study, conclusions cannot be drawn regarding each factor in relation to reading rate and accuracy.

Furthermore, the only study that looked at the effects of manipulating text presentation on reading comprehension, in children found that fonts with decorations (i.e., disfluent fonts) were comprehended as well as fonts without them [29]. However, based on the findings of the effects of font size and line length on rate and accuracy of sentence reading, we would except they may also influence reading comprehension. Such information may have far reaching applied implications. As reading comprehension required the orchestration of many subskills [38], and an interaction between reader and text, it is important to study the effects of text presentation beyond the reading speed and accuracy level. Thus while rate and accuracy are necessary for comprehension, they are not sufficient. Factors that influence them may influence comprehension in a different manner.

To summarize, creating less accessible perceptual presentation of text, or disfluent text (smaller fonts, less spacing) was found to have different effects on the reading speed and accuracy of skilled versus unskilled readers. In terms of size, larger font size enhanced reading speed and accuracy of younger and dyslexic readers and showed no effect on older children. In addition, it did not affect recall in older university students. However, bolding or italicizing text did improve long-term memory in older high school and university students. To the extent that text presentation affects reading rate and accuracy, we would expect it to influence reading comprehension as well. Thus, we hypothesized that for younger readers, manipulating text presentation by increasing disfluency compared to the standard text they are used to would impede comprehension, as they still receive important contextual cues from the print. For older children, who have already mastered the decoding and efficiency stages and thus rely less on actual visual cues, we hypothesized that increased disfluency (less familiar and accessibly text presentation) would function as a desirable difficulty, resulting in deeper processing and thereby increasing comprehension.

### The Current Experiments

In the current experiments, we examined the effect of perceptual fluency on reading comprehension in second and fifth grade children (Experiments 1 and 2, respectively). Specifically, we asked whether font size, line length, and line spacing would affect performance on a reading comprehension task. In addition, we asked whether these factors would differentially affect children in earlier versus later stages of reading development.

## Experiment 1: Second Grade

Experiment 1 was designed to examine how the perceptual disfluency of text, created by decreasing font size, increasing line length, and decreasing line spacing, affects reading comprehension among second graders.

### Method

#### Participants

Participants were 45 second graders (20 girls, mean age 7.5 years) from elementary schools in Israel, mostly of middle- and upper-middle-class socioeconomic background. All children had rapid naming, reading and verbal abilities in the average range, based on standardized measures [39,40].

The research conducted in this paper was approved by the university review board - The Ethics Committee Review Board-IRB. The members of the Helsinki committee in our university are Shoshi Zalka and Avi Karni. Informed written consent was obtained from the parents and children, also, the data were analyzed anonymously). Finally, the investigation was conducted according to the principles expressed in the Declaration of Helsinki.

#### Materials

To examine reading comprehension, we developed a tool that included four age-appropriate texts, matched for level of difficulty and length. The texts were adapted from previous national reading assessment materials and were 44-47 words long. We manipulated three dimensions between the texts: font size, line length, and spacing between lines. The dimensions of the baseline text—20 pt font size, 4.2 inch line length, double line spacing—represented the text dimensions that are used for national reading assessment for the second grade, and reflected the typical font size, line length, and line spacing used in textbooks for this grade. For the other three texts, we manipulated presentation by decreasing font size in one text by 20%; increasing line length in another text by 20%; and decreasing line spacing by 20% in the final text. The assignment of each specific text to one of the four dimensions, as well as the order of text presentation, was counterbalanced across participants. For ecological reasons, texts were presented to children in a booklet. The design was self paced, as to ensure children are reading in their natural pace that is most comfortable for them.

After reading each text, students were asked to answer four multiple choice reading comprehension questions that were developed especially for the current study. Prior to conducting the study, the texts and questions were given to 10 judges, all with master’s degrees in literacy, to assess that they are indeed age-appropriate. The reliability of the tool, as examined in a pilot study with 51 children, was high (Cronbach’s α = .756).

#### Procedure

The consent of the parents, the children, and of the school was obtained before beginning the study. Children were first tested individually in a quiet room at school to determine reading and vocabulary levels. Next, there was a group administration of the reading comprehension tool. Children were told they would be asked to read several passages and answer some questions about them. They had up to 30 minutes to complete the entire task.

### Results

**Figure 1:**
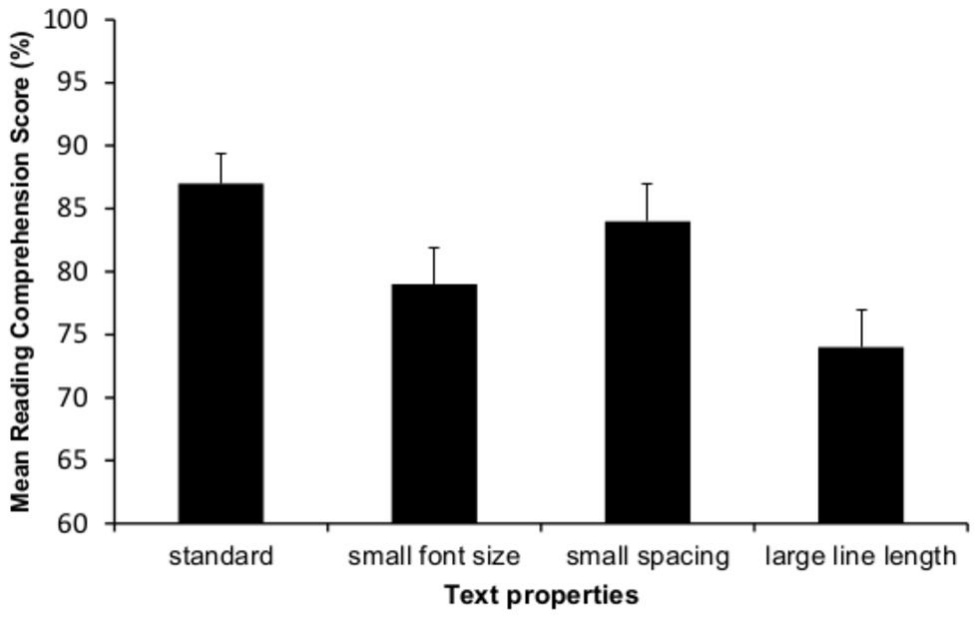
Mean reading comprehension score by text property for 2^nd^ grade (Experiment 1). Error bars designate +1SEM.

For each participant, we calculated an overall reading comprehension score for each text by computing the proportion of reading comprehension questions answered correctly out of four. Results are presented in Figure 1. The analysis yielded a significant effect of font size on reading comprehension: second graders had higher comprehension scores on the standard font-size text (.87) than the small font-size text (.79), *t*(43) = 2.32, *p*< .05, Cohen’s d = .35. The analysis also yielded a significant effect of line length: Second graders had higher comprehension scores on the standard font-size text than the large line-length text (.74), *t*(40) = 3.35, *p*< .01, Cohen’s d = .54. Comparing reading comprehension between the standard text and the small spacing text did not yield a significant effect, *t*(43) = 1.00, *ns*, Cohen’s d = .153.

## Experiment 2: Fifth Grade

The results of Experiment 1 support the prediction that for young children learning to read, increasing the perceptual disfluency of text, by decreasing font size or increasing line length, impairs comprehension. Experiment 2 was designed to examine the hypothesis that the opposite pattern, i.e., increased comprehension for more disfluent texts, would emerge for older children in fifth grade.

### Method

#### Participants

Participants were 45 fifth graders (24 girls, mean age 10.5 years) drawn from the same schools as the participants in Experiment 1. All children had reading and verbal abilities in the average range, based on standardized measures [39,40]. See Table 1.

**Table 1:** Descriptive statistics for PPVT, Rapid Naming, and Word Reading from Alef ad Taf for second and fifth grade children (N = 45 & 45, respectively).

	Second Grade Children		Fifth Grade children	
	Mean (SD)	Min-Max	Mean (SD)	Min-Max
PPVT SS	93.7 (5.2)	85-130	93.33 (3.2)	83-124
Word Reading SS	109 (5.1)	88-122	105 (6.9)	87-116
Naming Speed Raw	39 sec (8.6)	26-58	30.00sec (5.6)	18-42

#### Materials and Procedure

To examine reading comprehension, we developed a tool that was equivalent to the one used in Experiment 1 but adapted for fifth graders by employing the following changes: (1) the four age-appropriate texts were adapted from previous national reading assessment materials for the fifth grade and were 110-120 words long; (2) the dimensions of the baseline text represented the text dimensions that are used for national reading assessment for the fifth grade, and reflected the typical font size, line length, and line spacing used in textbooks for this grade—13 pt font size, 4.6 inch line length, one and a half line spacing. Again, disfluency was created for the other three texts by decreasing the font size of one text by 20%, increasing the line length of another text by 20%, and decreasing line spacing by 20% for the final text. The reliability of the tool, as examined in a pilot study with 50fifth graders, was high (Cronbach’s α = .797). The procedure was identical to the one used in Experiment 1.

### Results

**Figure 2:**
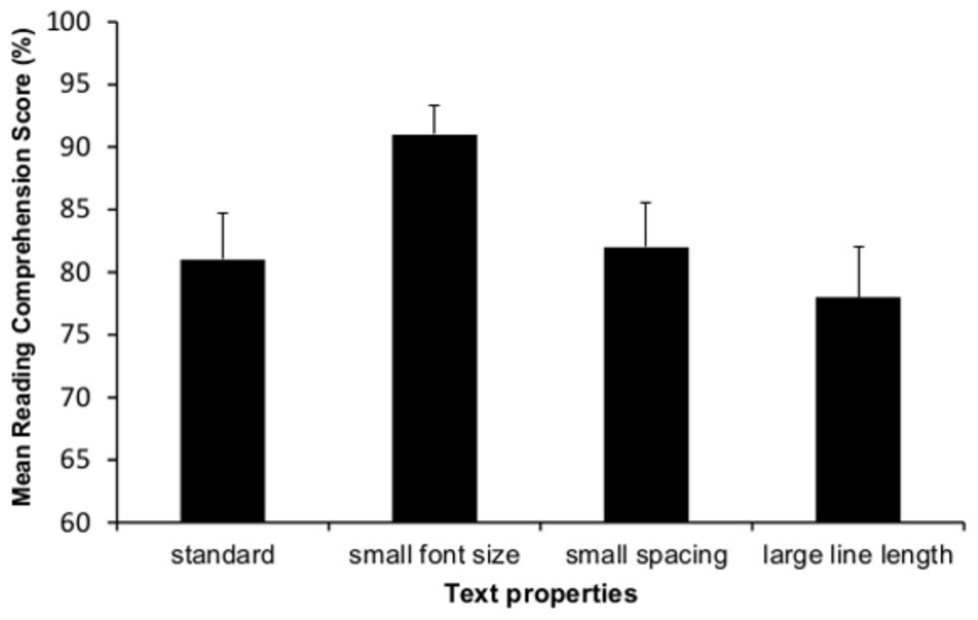
Mean reading comprehension score by text property for 5^th^ grade (Experiment 2). Error bars designate +1SEM.

For each participant, we calculated an overall reading comprehension score for each text by computing the proportion of reading comprehension questions answered correctly out of four. Results are presented in Figure 2. Results yielded a significant effect of font size on reading comprehension: in contrast to the effect found for second graders, fifth graders had higher comprehension scores for the *smaller* font-size text (.91) than the standard font-size text (.81), *t*(44) = -2.72, *p*< .01, Cohen’s d = .43. No significant effects of line length or line spacing were obtained, *t*(44) = .92, *ns*, Cohen’s d = .14 and *t*(44) = -.33, *ns*, Cohen’s d = .02, respectively.

To further examine the opposite effects of font size obtained for second graders in Experiment 1 and fifth graders in Experiment 2, we pooled the data across the two experiments and conducted a mixed-design analysis of variance, with font size (small vs. standard) and grade (second vs. fifth) as within- and between-participant factors, respectively. No main effects of font size or class were obtained, both *F*’s < 1. Importantly, the analysis yielded a significant 2-way interaction between font-size and class, *F*(1, 87) = 12.74, MSE = .45, *p*< .001, *η*_*p*_^2^ = .13. Thus, in fifth graders, comprehension benefited from decreasing font size, whereas in second graders, comprehension was impaired by this manipulation. Equivalent analyses yielded only a marginally significant interaction for line length, *F*(1, 84) = 3.26, MSE = .47, *p* = .07*, η*_*p*_^2^ = .04, and a non-significant interaction for line spacing, *F*< 1.

## General Discussion

Reading comprehension research has mainly focused on differences between good and poor comprehenders, and on the cognitive and linguistic factors that predict efficient extraction of meaning from text [38]. The current study represents a novel approach to research on reading comprehension by suggesting that, (1) merely manipulating the typographical aspects of text presentation can affect reading comprehension among children, and (2) children at different stages in the development of reading respond differently to these typographical manipulations. Specifically, the results of the current experiments suggest that manipulating presentation of text and making it disfluent has opposite effects on children at different reading stages. Among young readers in second grade, reading comprehension became more impaired when a text was made less fluent by decreasing font size or increasing line length, whereas in fifth grade, reading comprehension benefited from the increased disfluency brought on by decreased font size. Interestingly, line spacing did not have an effect on both grades, indicating that perhaps it is not a contextual cue that children rely on in retrieving information.

In the following sections, we address two theoretical issues. First, we suggest that current theoretical and applied models of reading comprehension should be expanded to include text presentation/perceptual fluency as a factor of influence. Second, we discuss the notion of "desirable difficulties" in learning [17,19] from a developmental perspective, emphasizing its implications for reading instruction.

### The Effect of Manipulating Text Presentation on Reading Comprehension

The opposite substantial effects of text presentation on reading comprehension for younger vs. older children have theoretical and educational implications. Theoretically, they suggest that the interaction between the reader and the text is not only content based, nor does it solely relate to factors such as background knowledge, proficiency in decoding, spelling etc.

In fact, text presentation changes the way information is encoded and processed. This notion has been previously suggested for reading rate and accuracy of young children. In the current research we suggest that similar effect exists for higher level processing (i.e., reading comprehension), and also for older children. Along similar lines with previous studies that have shown that decreasing font size impairs reading rate and accuracy in young children, in this study decreasing font size impaired comprehension. However, for older children, previous studies showed that decreasing font size did not affect their reading rate and accuracy [34,37], whereas in this study it actually *enhanced* comprehension.

In order to understand the mechanism that may underlie the different effects on younger vs. older children we suggest a distinction between two bases of reading-related fluency. The first stems from differences in the reader component of the RAND model [14] and is the traditional *reading fluency* [41]: the rate, accuracy, and proficiency of reading. The second stems from differences in the text component of the RAND model is what we have termed *perceptual fluency*, which is affected by be manipulations of perceptual features of text presentation.

### Reader-Based Fluency Differences

The direction of causality between reading fluency and comprehension is currently a matter of some debate [41]. There is evidence that reading fluency both contributes to and is a product of comprehension [42]. These researchers thus advocated viewing comprehension and (reading) fluency as having a reciprocal causal relationship, a view currently espoused by practitioners as well as reading researchers [43]. Traditionally, however, researchers have theorized that reading fluency primarily facilitates comprehension, in line with automaticity theory [5]. Thus, the more fluent a reader is, the more resources could be allocated for comprehension. The assumption behind the automaticity theory is that memory can only cope with the demands needed for reading if important components can be processed automatically [44]. It is believed that the relationship between reading fluency and comprehension depends, to some degree, on reading skill [45]. It has been found that reading fluency was less strongly correlated with reading comprehension among poor readers with an isolated reading fluency (speed) deficit than it was among more proficient readers [7]. That is for some poor slow readers, spending more time may be an effective strategy. Hence better readers are typically faster and more accurate. However, slow readers in some case could have adequate comprehension, while some struggling comprehenders are typically slow, and laborious [46]. Many reading interventions in fact have focused on reading acceleration as a means towards enhancing comprehension [47, 48].

### Text-Based Fluency Differences

The current study postulates a supplementary view of the relationship between fluency and comprehension. We suggest that under certain conditions, manipulating text-based fluency (perceptual fluency), making text more or less easy to process, may actually, and counterintuitively, enhances comprehension, at least for older, upper elementary school students.

Thus, in the context of the RAND model of comprehension, which includes three components, the reader, the text, and the activity, we suggest that the text component should address not only the text type, but also the typographical text presentation mode [14].

In the current study, older readers who had mastered the efficiency stage of reading benefited from a decrease in font size that made the print *less perceptually fluent*. Presumably, the decreased perceptual fluency made the text more difficult to process, at least subjectively, leading them to engage more deeply in reading the text. Similar to previous studies that examined only rate and accuracy of sentence reading and not comprehension, children in second grade, who had not yet reached efficiency, did not benefit from the manipulation of text or the decrease in fluency [40]. This may have caused them to read slower, thus impacting their memory for what they read [49]. However the older children, who naturally were faster, benefited from the added difficulty of processing smaller text. Findings from the current study suggests that only after lower level skills have been mastered, such that there is indeed a relationship between reading fluency and comprehension, does perceptual disfluency become an effective mean for reaching deeper processing (improved comprehension). In younger children, who are still developing the concurrent sub-skills involved in reading (decoding, speed, orthography, etc.), the increase in cognitive load created by disfluent text and the mental effort required for comprehension do not result in better performance. Interestingly, the extra spacing effect that was found to aid dyslexic readers, did not aid the young children in this study. Similar to adults For instance, reading speed in skilled adult readers is slowed when letter spacing is doubled [3].

Since we did not time the children, as this was a silent reading task, we do not know if this was due to the prolonged time they spent on the task, or their altered strategies in interacting with the text that changed their reading scores. However, measures that tap reading time, such as rapid naming and timed word reading did not correlate with any of the reading comprehension text conditions indicating that slow and fast readers were similarly affected by the task. Future studies should also examine reaction times for reading text in different fonts.

### Developmental Perspective on Desirable Difficulties

As noted in the introduction, numerous studies in cognitive psychology have shown that manipulations that introduce difficulties during learning often enhance learning, somewhat counterintuitively [17]. However, it is important to emphasize that not all difficulties are desirable [50]. As noted, “Many difficulties are undesirable during instruction and forever after. Desirable difficulties, versus the array of undesirable difficulties, are desirable because they trigger encoding and retrieval processes that support learning, comprehension, and remembering. If, however, the learner does not have the background knowledge or skills to respond to them successfully, they become undesirable difficulties.” [19]. To design instruction optimally, educators should therefore be able to choose the appropriate level of difficulty to support learning, rather than hinder it.

The current study contributes to this common view of difficulties as desirable to learning by raising the need to consider developmental trends when determining the difficulty levels used during instruction. For example, French at his colleagues, found that making fonts disfluent had greater effects on students with dyslexia than on typical adolescent readers [49] In the current study, increasing perceptual disfluency by changing typographical features of the text from the standard children are accustomed to, enhanced comprehension for older, skilled readers, but impaired comprehension for young, unskilled readers. Clearly, the standard text presentation format was difficult enough for the younger children, such that added difficulty was detrimental to them. Thus, to promote learning, specific instruction conditions should be carefully examined in light of the students’ skill level, and educators should keep in mind that the optimal level of difficulty to support learning is constantly changing as children develop their learning skills.

### Future Directions and Concluding Comments

Our results provide an initial, unique exploration of the effect of manipulating text features and on reading comprehension, though more research is needed to achieve a more comprehensive understanding of this process. While better comprehenders are often more fluent as readers, when looking at the text component, making the text (as opposed to the reader) less fluent may be more beneficial for instruction of older readers. In this study, in order to ensure ecological validity we did not control for reading time. Future studies should examine whether enhanced comprehension is due to prolonged reading in any of the conditions. However, reading time might not have played a role in the findings, as previous studies indicate that smaller font sizes did not affect the reading speed of older children [29]. To better understand our results, future studies may also directly examine how readers monitor their comprehension and learning of text while reading, and how disfluency affects such monitoring by, for example, eliciting online judgments of learning. In addition, it is important that future research examine not only the effect of perceptual fluency on online processing of text, but also its effect on delayed retention of the information read. Finally, it would be interesting to examine if any of the conditions has an effect of motivation.

In conclusion, indeed one should not judge a book by its cover, but one should pay attention to how the text is presented within. This is especially important today, when many texts are read using electronic devices and font size can be easily manipulated.
